# Comparison of subjective quality of life after endoscopic submucosal resection or surgery for early gastric cancer

**DOI:** 10.1038/s41598-020-62854-7

**Published:** 2020-04-21

**Authors:** Chung Hyun Tae, Ki-Nam Shim, Byung-Wook Kim, Jie-Hyun Kim, Su Jin Hong, Gwang Ho Baik, Hyun Joo Song, Yong Sung Kim, Seung-Ho Jang, Hye-Kyung Jung

**Affiliations:** 1 Department of Internal Medicine, Ewha Womans University College of Medicine, Ewha Medical Research Institute, Seoul, Republic of Korea; 2 Division of Gastroenterology, Department of Internal Medicine, Incheon St. Mary’s Hospital, College of Medicine, The Catholic University of Korea, Seoul, Republic of Korea; 3 Department of Internal Medicine, Gangnam Severance Hospital, Institute of Gastroenterology, Yonsei University College of Medicine, Seoul, Republic of Korea; 4 Digestive Disease Center and Research Institute, Department of Internal Medicine, Soonchunhyang University College of Medicine, Bucheon, Republic of Korea; 5 Institute for Liver and Digestive Diseases, Department of Internal Medicine, Hallym University College of Medicine, Chuncheon, Republic of Korea; 6 Department of Internal Medicine, Jeju National University School of Medicine, Jeju, Republic of Korea; 7 Wonkwang Digestive Disease Research Institute, Wonkwang University, Iksan, Republic of Korea; 8 Department of Psychiatry, School of Medicine, Wonkwang University, Iksan, Republic of Korea

**Keywords:** Cancer, Gastroenterology

## Abstract

Quality of life (QoL) has become an important issue after early gastric cancer (EGC) treatment. We aimed to compare the QoL of EGC survivors after ESD (n = 241) or laparoscopic subtotal gastrectomy (n = 241) without recurrence and to evaluate the QoL over the 5-year period after adjusting for various confounding factors related to QoL. QoL related to the gastric cancer subscale (GCS) was significantly higher in the ESD group than surgery group (*p* < 0.001). After adjusting for all possible confounding factors, survivors who underwent ESD still had higher QoL related to CSG than those who underwent surgery. On the analysis of interaction effects for all QoL subscales, higher QoL related to GCS of ESD group than those of surgery group has been kept over time (*p* = 0.983). Therefore, we concluded that EGC survivors who undergo ESD have significantly better QoL related to GCS over a 5-year period after treatment than those who undergo surgery. This may be a useful consideration when selecting treatment modalities for patients with EGC.

## Introduction

Based on the 2015 nationwide cancer statistics, gastric cancer (GC) is the most prevalent type of cancer and is the fourth most common cause of cancer mortality in Korea^1^. On implementation of GC screening as part of the National Cancer Screening Program in 1999, screening rates of GC have increased from 7.5% in 2002 to 47.3% in 2012^2^. Up to 75% of total GC had early gastric cancers (EGCs). Similarly, the proportion of EGCs treated with surgery has increased over time, accounting for 61% of all GCs in 2014 based on a nationwide surgery database^3^. High 5-year survival rates and increased long-term survival of patients with EGCs have led to concerns about the quality of life after the complete cure of EGCs. Maintaining gastric function, minimizing post-operative complications, and maximized improvements of quality of life (QoL) have become important issues related with surgery. Recently, minimally invasive surgical approaches and function-preserving surgeries are commonly performed in patients with EGCs. The total laparoscopic approach, which is one of the minimally invasive surgical approaches, ranked first according to the nationwide data of surgery in 2014^3^. Function-preserving gastrectomy, both pyloric-preserving gastrectomy and proximal gastrectomy have been performed with curative intent in patients with EGCs^4^. However, in spite of these efforts, patients who inevitably undergo surgical resection would face certain limitations of overall gastrointestinal function. A lot of sequelae, such as early satiety, loss of appetite, heartburn, dysphagia, nausea, vomiting, dumping syndrome, and weight loss have a profound impact on QoL in these patients^5^.

In contrast, the role of endoscopic submucosal dissection (ESD) in EGC patients at low risk for lymph node metastasis also has been steadily increasing. Many western studies comparing ESD to surgery have reported comparable oncological safety and efficacy in cases of clear resection of EGCs, with low rates of procedure-related complications, high cure rates, and extremely infrequent metastatic recurrence, even in elderly patients with the poor general condition^6–9^. With these outstanding short and long-term outcomes of ESD, it is becoming an acceptable treatment for EGCs in patients with specific indications. If oncological outcomes do not differ between ESD and surgery, treatment option to achieve better QoL should be selected for EGC patients. So far, only two studies have compared the difference of QoL between ESD and surgery: One cross-sectional study demonstrated a better QoL related to symptomatic and gastrointestinal function in the ESD group^10^. The other study with the follow-up of 24 months after each treatment demonstrated that ESD can provide better QoL in terms of physical functioning, body image, and stomach itself symptomatic problem^11^. However, these studies didn’t demonstrate whether the superior QoL would be sustained or would disappear over time. In addition, they didn’t consider various confounding factors impacting QoL.

Previous research has suggested that psychological personality factors substantially affect well-being and have positive or negative impacts on disease recovery in patients, and may lead to impaired QoL and mental health status^12–14^. Cardiac patients with high levels of anxiety, depression, and lower levels of satisfaction with life have poor psychological well-being^13^. Cancer survivors with Type D personality rather than non-type D personality had more pain and general discomfort^12,14^. While poor sleep quality, insomnia, and inadequate sleep time reduced the QoL^15^, high degrees of resilience contributed to a good QoL in cancer survivors^16^.

We aimed to demonstrate whether EGC survivors who underwent ESD have a superior QoL compared to those who underwent surgery, after adjusting for various confounding factors including socio-demographic characteristics and psychological factors that could influence the QoL. Second, we evaluated whether the QoL differences between the two groups would remain over time.

## Results

### Study population characteristics

Baseline characteristics of the study population are summarized in Table 1. Mean ages of ESD group at both enrollment and treatment time point were older than the surgery group (both *p* < 0.001). The proportion of men and women was not different in both groups.

**Table 1:** Baseline characteristics between ESD and surgery groups.

	ESD group (n = 241)	Surgery group (n = 241)	*P* value
Age (years), mean ± SD			
At enrollment	64.4 ± 9.2	58.2 ± 10.8	<0.001
At treatment	62.2 ± 9.4	55.7 ± 10.9	<0.001
Sex, n (%)			0.565
Men	162 (67.2)	155 (64.3)	
Women	79 (32.8)	86 (35.7)	
Histology, n (%)			<0.001
Differentiated	228 (94.6)	108 (44.8)	
Undifferentiated	13 (5.4)	133 (55.2)	
Tumor size (mm)	15.6 ± 12.8	23.9 ± 16.1	<0.001
Invasion depth, n (%)			<0.001
Mucosa	225 (93.4)	163 (67.6)	
Submucosal	16 (6.6)	78 (32.4)	
Adverse event after treatment, n (%)			0.015^a^
Early	16 (6.6)	5 (2.1)	
Late	1 (0.4)	0	
Elapse time since ESD or surgery, mean ± SD (year)	2.1 ± 1.5	2.5 ± 1.6	0.011
3 month–1 year, n (%)	76 (31.5)	68 (28.2)	0.274
1–2 years, n (%)	52 (21.6)	39 (16.2)	
2–3 years, n (%)	42 (17.4)	43 (17.8)	
3–4 years, n (%)	40 (16.6)	47 (19.5)	
4–5 years, n (%)	31 (12.9)	44 (18.3)	
Resection type			
Distal gastrectomy	—	238 (98.8)	
Proximal gastrectomy	—	3 (1.2)	

^a^*P* for Fisher’s exact test.

Tumor characteristics differed between the groups. The EGC group that underwent ESD had tumors with mostly differentiated histology (*p* < 0.001), smaller size (*p* < 0.001), and less deep invasion (*p* < 0.001), resulting from the difference of standard treatment strategy according to the tumor characteristics.

Early adverse events after treatment were more frequently reported in the ESD group: post-ESD bleeding (n = 12) was most frequent, followed by micro-perforation (n = 3) and electrocoagulation syndrome (n = 1). The surgery group had experienced 5 early adverse events; namely, anastomotic leakage (n = 1), postoperative pneumonia (n = 1), postoperative fever (n = 1), acute renal failure (n = 1), and severe postoperative nausea and vomiting (n = 1). In addition, there was one late adverse event of pyloric stenosis after ESD. However, no late adverse event was reported in the surgery group.

Classified by elapsed time (per year) since ESD or surgery, there were no differences in the proportions of the study population between ESD and surgery groups (*p* = 0.274). However, the mean elapsed time of the ESD group was shorter than the surgery group (*p* = 0.011).

With respect to socioeconomic and general health status, both groups showed approximately even distribution of the employment status, living with a partner, religion, income, current smoking, and family history of GC, as shown in Table 2. However, the surgery group had a higher education level (*p* = 0.003), fewer alcohol drinking (*p* = 0.010), and fewer co-morbidities (*p* < 0.001), but also a poorer ECOG performance status at the time of enrollment (*p* < 0.001) than the ESD group.

**Table 2:** Socioeconomic and general health status between ESD and surgery groups.

	ESD group (n = 241)	Surgery group (n = 241)	*P* value
Employment, n (%)			0.141^a^
Full or part-time	115 (47.7)	125 (51.9)	
Unemployed	70 (29.1)	52 (21.6)	
Housewife	56 (23.2)	62 (25.7)	
Student	0	2 (0.8)	
Living with partner, n (%)			0.180
Yes	77 (73.3)	63 (63.6)	
No	28 (26.7)	36 (36.4)	
Religion, n (%)			0.521
Present	130 (53.9)	138 (57.3)	
Absent	111 (46.1)	103 (42.7)	
Education level, n (%)			0.003
Middle school graduate or below	110 (45.6)	74 (30.7)	
High school graduate	82 (34.0)	103 (42.7)	
College graduates or above	49 (20.3)	64 (26.6)	
Income (KRW)		C	0.465
<200	100 (41.5)	87 (36.1)	
200–400	98 (40.7)	109 (45.2)	
>400	43 (17.8)	45 (18.7)	
Current smoking, n (%)	38 (15.8)	28 (11.6)	0.233
Alcohol drinking, n (%)	86 (35.7)	59 (24.5)	0.010
Current EGOC performance status			<0.001
1	213 (88.4)	175 (72.6)	
≥2	28 (11.6)	66 (27.4)	
Number of co-morbidities, median (min-max)^b^			
0	107 (44.4)	164 (68.1)	<0.001
1	87 (36.1)	55 (22.8)	
≥2	47 (19.5)	22 (9.1)	
Family history of gastric cancer, n (%)	69 (28.6)	78 (32.4)	0.429

^a^*P* for Fisher’s exact test.

^b^Included hypertension, diabetes mellitus, cardiovascular disease (congestive heart disease, ischemic heart disease, heart failure), chronic liver disease (liver cirrhosis, choric, renal disease, psychologic disorders, pulmonary disease, thyroid function disorders, joint disease (arthritis, rheumatism), sleep disorders, or self-reported chronic diseases.

There were significant differences in Distress Thermometer and Cancer Worry Scale scores between the two groups (Table 3). The surgery group had a higher Distress Thermometer scale score (*p* = 0.016). However, there was no significant difference of clinically significant distress level (*p* = 0.162). Patients who underwent ESD reported that their physicians, during their medical appointments, seem to worry about the recurrence of their cancer (*p* = 0.049). However, there were no differences between the groups with regard to general worry about cancer by themselves, cancer-related concerns, symptoms, and overall scores for worry about cancer recurrence. In addition, no significant differences between groups were observed for the HADS, Type D personality scale, Connor-Davidson resilience scale, and Pittsburgh sleep quality index scores.

**Table 3:** Psychometric characteristics between ESD and surgery groups.

Variables, mean ± SD (median)	ESD group (n = 241)	Surgery group (n = 241)	*P* value
HADS			
Anxiety	2.5 ± 3.0 (1.0)	2.8 ± 3.2 (2.0)	0.258
Depression	3.2 ± 3.4 (2.0)	3.7 ± 3.4 (3.0)	0.162
Distress thermometer	1.6 ± 2.0 (1.0)	2.1 ± 2.5 (1.0)	0.016
>4	202 (83.8)	189 (78.4)	0.162
≤4	39 (16.2)	52 (21.6)	
Type D personality scale			
Negative affectivity	4.5 ± 5.2 (3.0)	4.9 ± 5.5 (3.0)	0.343
Social inhibition	4.8 ± 5.2 (3.0)	5.5 ± 5.2 (4.0)	0.134
Connor-Davidson resilience scale	69.3 ± 18.2 (70.0)	70.8 ± 19.1 (72.0)	0.365
Pittsburgh sleep quality index	4.6 ± 2.9 (4.0)	4.6 ± 3.1 (4.0)	0.976
Cancer Worry Scale scores			
Patient recur worry	1.9 ± 2.1 (1.0)	1.9 ± 2.1 (1.0)	0.732
Doctor recur worry	1.8 ± 2.2 (0.0)	1.4 ± 1.9 (0.0)	0.049
Concern for some months	1.5 ± 1.8 (0.8)	1.5 ± 1.8 (0.8)	0.861
Symptom	1.4 ± 1.6 (0.8)	1.6 ± 1.7 (1.3)	0.107
Total score	6.5 ± 6.0 (5.7)	6.3 ± 5.5 (5.3)	0.668

Abbreviations: HADS, the Hospital Anxiety and Depression Scale, which consists of 7 items for the anxiety and 7 items for the depression subscales.

### Quality of life comparison between the ESD and surgery groups

The results shown in Table 4 demonstrate the QoL comparison between the ESD and surgery groups by univariate analyses and ANCOVA adjusted for confounders.

**Table 4:** Adjusted QoL items between ESD and surgery groups.

QoL items	Univariate analyses mean ± SD (median)		*P* value	ANCOVA Model 1^a^		*P* value	ANCOVA Model 2^b^		*P* value
							Least square mean (95% CI)		
	ESD group (n = 241)	Surgery group (n = 241)		Least square mean (95% CI)	Surgery group		ESD group	Surgery group	
PWB subscale (7 items)	3.7 ± 0.4 (3.9)	3.6 ± 0.5 (3.8)	0.029	3.7 (3.7–3.8)	3.6 (3.5–3.7)	0.011	3.6 (3.5–3.6)	3.5 (3.5–3.6)	0.385
SWB subscale (7 items)	2.3 ± 0.9 (2.4)	2.3 ± 1.1 (2.3)	0.632	2.3 (2.2–2.5)	2.3 (2.1–2.4)	0.459	2.4 (2.2–2.5)	2.3 (2.1–2.4)	0.421
EWB subscale (6 items)	3.4 ± 0.6 (3.5)	3.3 ± 0.7 (3.5)	0.536	3.3 (3.2–3.4)	3.3 (3.2–3.4)	0.944	3.1 (3.0–3.2)	3.2 (3.1–3.3)	0.282
FWB subscale (7 items)	2.8 ± 0.8 (2.9)	2.8 ± 0.9 (2.9)	0.616	2.8 (2.7–2.9)	2.7 (2.6–2.8)	0.301	2.7 (2.6–2.9)	2.7 (2.6–2.9)	0.901
Gastric cancer subscale (19 items)	3.5 ± 0.4 (3.6)	3.3 ± 0.5 (3.5)	<0.001	3.5 (3.5–3.6)	3.3 (3.2–3.3)	<0.001	3.4 (3.3–3.5)	3.2 (3.1–3.3)	<0.001
FACT-Ga total ^c^	15.8 ± 2.1 (16.0)	15.3 ± 2.7 (15.6)	0.035	15.6 (15.3–16)	15.1 (14.8–15.5)	0.029	15.1 (14.8–15.5)	14.9 (14.5–15.3)	0.321

Abbreviations: QoL, quality of life; SD, standard deviations; CI, confidence interval; PWB, physical well being; SWB, social well being; EWB, emotional well being; FWB, functional well being; FACT‐G, FACT‐Ga, Functional Assessment of Cancer Therapy‐Gastric quality‐of‐life instrument.

^a^ANCOVA Model 1 was adjusted by the age at enrollment and sex.

^b^ANCOVA Model 2 was adjusted by age at enrollment, sex, years since treatment, education level, alcohol drinking, current ECOG performance status, number of co-morbidities, Distress Thermometer, and doctor’s recurrence worry in Cancer Worry Scale.

^c^FACT-Ga total is calculated as the sum of PWB, SWB, EWB, FWB, and Gastric cancer subscale.

In the univariate analyses, ESD groups had higher scores of PWB (*p* = 0.029) and GCS (*p* < 0.001). Also, FACT-Ga total (*p* = 0.035), which is the aggregate of PWB, SWB, EWB, FWB, and GCS scores, were also higher in the ESD group.

To rule out the possibility of effect of confounder factors, ANCOVA was performed in Model 1 (adjusted for age at enrollment and sex) and Model 2 (adjusted for age at enrollment, sex, years since treatment, education level, alcohol drinking, current EGOC performance status, number of co-morbidities, Distress Thermometer, and doctors’ recurrence worry in Cancer Worry Scale). In ANCOVA Model 1, PWB (*p* = 0.011), GCS (*p* < 0.001), and FACT-Ga total score (*p* = 0.029) remained significant after adjustment for age and sex. In ANCOVA Model 2, after adjustment for all possible confounding factors, only QoL related to the GCS was significantly higher in the ESD group (*p* < 0.001). There was no difference in QoL related to PWB, SWB, EWB, and FWB after adjustment for confounding factors.

### Difference of quality of life according to the elapsed time after ESD or surgery

**Figure 1:**
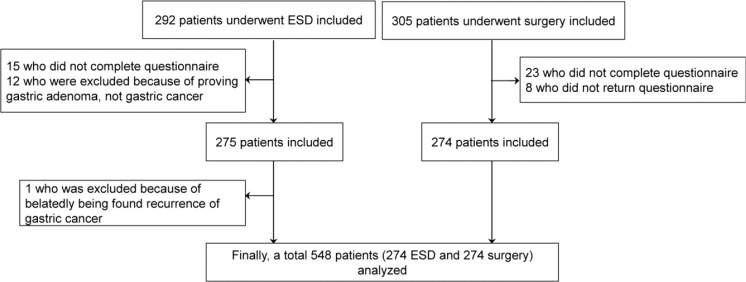
Time × Group interaction effects on QoL related to gastric cancer subscale. It reflected that significant difference of QoL related to gastric cancer subscale known through ANCOVA analysis demonstrated no discernable change across time (*p* = 0.983).

To test whether higher QoL related to GCS changes over time between ESD and surgery groups, we used the Time × Group interaction effects after adjusting age at enrollment, sex, education level, alcohol drinking, current ECOG performance status, number of co-morbidities, distress thermometer, and doctor’s recurrence worry. It revealed that higher QoL related to GCS of ESD group than those of surgery group has been kept over time (*p* = 0.983), as shown in Fig. 1.

In addition, to test whether comparable QoL related to PWB, SBW, EWB, and FWB in two groups changes over time, we performed the Time × Group interaction effects after adjusting the variables listed above. QoL related to PWB, SBW, EWB, and FWB between two groups was similar to the surgery group over time (*p* = 0.410 for PWB, *p* = 0.662 for SWB, *p* = 0.907 for EWB, and *p* = 0.535 for FWB).

## Discussion

Based on the accumulating data on comparable outcomes of ESD and surgery in terms of curability, 5-year survival rates, and adverse events, ESD is considered an optimal treatment for patients with EGC without risk of lymph node metastasis. In addition, the issue has been raised with improving QoL in long-term survivors after complete cure of EGCs. Clinicians have reasonably believed that stomach-preserving ESD might be superior to surgery in terms of QoL. However, few studies have directly compared patients’ QoL after adjusting for confounding factors to effect on QoL^10^.

Our findings support that ESD group is associated with better QoL related to the GCS of ESD than those of surgery group. For more objective assessment of individuals’ QoL and comparison of QoL between the two groups, we considered a wide range of personality factors and personal stressors such as underlying anxiety and depression, recent distress, type D personality of negative affectivity and social inhibition, resilience, quality of sleep, and worry of cancer recurrence, based on the evidence that these factors could affect QoL. It is well known that there is a close relationship between QoL and anxiety, depression, and emotional functioning in cancer survivors^17^. Most cancer patients and survivors experience intense anxiety and depression, which negatively affect QoL^18^. In particular, the prevalence of depression in GC survivors is known to be high even after being disease-free^19^. GCs survivors with depression were approximately 4.5 times more likely to develop insomnia^19^. Distress is particularly associated with poor social-emotional function affecting QoL^18^. Patients with both Type D personality and GC demonstrated increased levels of pain and fatigue affecting functional capacity and impairing QoL^12^. In addition, patients with low resilience have high psychological distress and low mental health-related QoL. GC patients who underwent ESD reported feeling that their physicians worry about recurrence of their cancer, which was an independent risk factor of poor QoL^10^. After adjusting for this wide range of personality types and personally stressful situations, we conclude that there is a difference in QoL related to the GCS between ESD and surgery groups. To the best of our knowledge, our study is the first attempt to compare QoL in EGC survivors who underwent either ESD or surgery after adjusting for this broad a spectrum of confounding personality types and personally stressful situations.

The GCS demonstrating a clear distinction between the two groups in our study consists of 19 items for assessment of patient-reported general symptoms, gastrointestinal symptoms, and lifestyle changes related to gastrointestinal function. In our study, patients who underwent surgery suffered from more severe digestive problems and related lifestyle changes than those underwent ESD. This had an intensely negative effect on QoL related to the GCS. Although surgery has been considered a preferred treatment with respect to certain treatment outcomes, inevitable sequelae such as postoperative weight loss, loss of appetite, dumping syndrome, anemia, gut hormonal change, and others occur.

To date, two studies have described QoL in patients who underwent either ESD or surgery^10,20^. However, those investigators used the European Organization for Research and Treatment of Cancer questionnaire, which is different from the one we used. In addition, all of the mean elapsed time since treatment in the enrolled subjects was also shorter than those in our study (maximum 18 months, 20.6 months). Similar to our results, their analysis of the surgery group showed more troublesome symptoms and a poorer body image than those of the ESD group^10,20^. However, PWB and FWB was also significantly better in the ESD group, as reported by Kim YI and colleagues, which is different from our results^20^. In our study, univariate analyses showed that PWB in ESD group seems to be superior as well. However, PWB didn’t significantly differ between the two groups on multivariate analysis after adjusting for a wide variety of variables. The reasons for the differing results among studies might be the lack of adjusted variables in QoL analyses and the different lengths of elapsed time since treatment.

We demonstrated that the QoL advantage of ESD over surgery according to the GCS was sustained up to 5 years post-treatment. Irrespective of either ESD or surgery, previous studies have shown that impaired QoL is inevitable immediately after treatment. According to a study of QoL in patients who underwent ESD, global health-related QoL and GCS QoL slightly deteriorated immediately after ESD^21^. In a study involving serial QoL assessment of patients who underwent surgical resection, negative impact on QoL typically occurred for at least 12 postoperative months and subsequently resolved^22,23^. However, different patterns of QoL recovery have been reported after ESD and surgery. Symptom and functional scale scores reportedly deteriorated immediately in patients with EGC after ESD, recovered compared with baseline within 6 months of follow-up, and remained unchanged thereafter^21,24^. However, most studies show that treatment-related symptom scores do not fully recover compared with baseline after surgical resection^11,21^. To date, there is only one study involving serial follow-up of QoL for 24 months post-treatment^20^. According to the results of that study, EGC patients who underwent ESD demonstrated better PWB and FWB, fewer stomach-related symptoms, and body image-related QoL benefits for 1 to 2 years post-treatment^20^. Although the results of our study are not based on longitudinal serial follow-ups, an absolute difference in the QoL of patients who underwent ESD compared to that of patients who underwent surgery could be confirmed for 3 months to 5 years post-treatment.

This study has some limitations. One weakness was that the cross-sectional study design didn’t allow the analysis of repeated measurement of individuals’ QoL over time. Second, selection bias cannot be fully excluded despite the consecutive enrollment of eligible subjects. Further study through rigorously designed randomized controlled trials is warranted. Second weakness was that patient’s attitude and way of thinking might be influenced their choice of EGC treatment modality, either EGC or surgery. Although the known various confounding factors were adjusted in our study, attention must be paid to the interpretation of our study.

Despite these limitations, we conclude that ESD and surgery’s differences in QoL related to GCS exists and is maintained over the 5-year postoperative period. Surgeons and clinicians should consider these differences of post-procedural QoL in EGC survivors after ESD or surgery if both treatment modalities are available.

## Methods

### Design and subject recruitment

This was the multicenter cross-sectional study conducted from December 2015 to June 2017 at five referral hospitals in the Republic of Korea providing both ESD and laparoscopic-assisted gastrectomy for EGC. The patients had undergone either ESD or laparoscopic-assisted gastrectomy according to the physician’s clinical judgment at that time and visited their gastroenterologist or surgeon for follow-ups.

Patients were eligible if they were at least 18 years old, the elapsed time was between 3 months and 5 years after ESD or laparoscopic-assisted gastrectomy for EGCs, and there was no evidence of recurrence at enrollment. The inclusion criteria for the ESD group were as follows: 1) *en bloc* resection with free vertical and lateral margins, and 2) tumor confined to the submucosal layer (<500 μm) without lymphovascular invasion and lymph node metastasis. The inclusion criteria for the laparoscopic-assisted gastrectomy (surgery) group were as follows: 1) *en bloc* resection without residual tumor, and 2) tumor confined to the submucosal layer with no lymphovascular invasion and no lymph node metastasis on extended D2 lymphadenectomy. Based on the AJCC 8^th^ TNM stage, TNM stage of ESD group and surgery group was all stage IA^25^.

**Figure 2:**
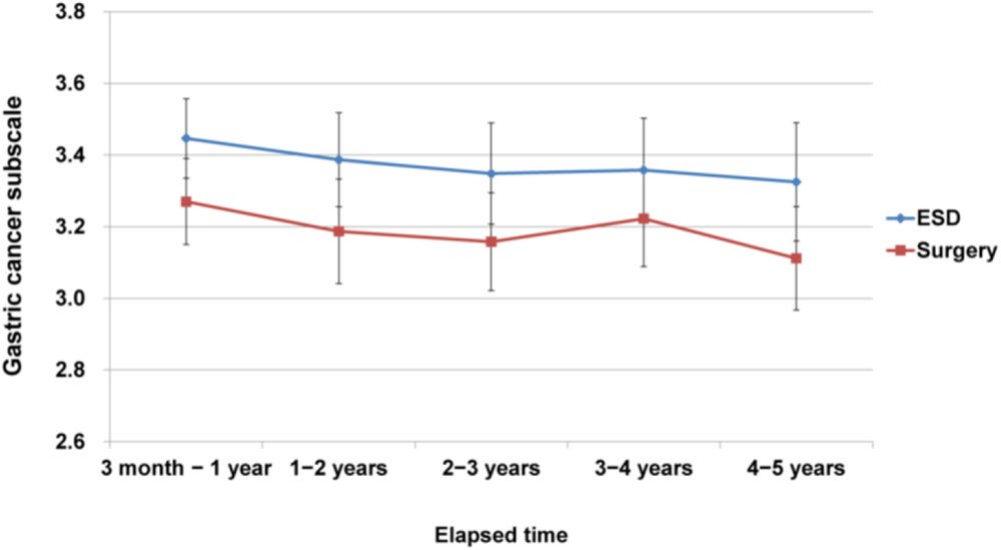
Flow diagram of enrolled participation.

We speculated that up to 3 months after ESD or laparoscopic-assisted gastrectomy, patients would still have troublesome treatment-related gastrointestinal symptoms resulting in unstable QoL, and those who didn’t show any evidence of recurrence for more than 5 years would have stable QoL. Therefore, we excluded patients who were within 3 months of or more than 5 years status-post ESD or laparoscopic-assisted gastrectomy. We also excluded patients who underwent total gastrectomy and near total gastrectomy because the loss of reservoir function is predictable as the result of inevitable worsening of QoL (Fig. 2).

This study was approved in all participating institutes (Ewha Womans University Mokdong Hospital Institutional Review Board, Institutional Review Board of Incheon St. Mary’s Hospital, Yonsei University Health System, Severance Hospital, Institutional Review Board, Soonchunhyang University Bucheon Hospital Institutional Review Board, Hallym University Chuncheon Sacred Heart Hospital Institutional Review Board, Jeju National University Hospital Institude Review Board, Wonkwang University Hospital Institutional Review Board, and Institutional Review Board Wonkwang University Sanbon Hospital). All subjects provided informed written consent before participating. This study has been carried out in accordance with the Declaration of Helsinki.

### Data collection

We collected the data of treatment and histopathological characteristics by reviewing the institutional database, which contained information about the tumor size (maximal tumor size and invasion depth), histological type (‘differentiated’ including well- and moderately-differentiated tubular adenocarcinoma and papillary carcinoma, and ‘undifferentiated’ including poorly differentiated adenocarcinoma, mucinous adenocarcinoma, signet ring cell carcinoma, and lymphoepithelioma-like gastric carcinoma), and time to treatment.

Complications were analyzed considering the onset time of complication after treatment. An early adverse event was defined as a complication that occurred within 3 months of treatment, while late complications were defined as occurring beyond 3 months of treatment. Post-ESD bleeding is defined as hematemesis or melena requiring transfusion with clinical evidence ESD-induced ulcer bleeding. The electrocoagulation syndrome is clinically defined localized abdominal pain, rebound tenderness, fever > 38 °C, and signs of peritoneal irritation without frank perforation. All ESD-related perforations was divided into a macroperforation and a microperforation. A macroperforation was defined as a gross perforation that occurred during an ESD procedure and a microperforation was defined by free air visible on X-rays after the procedure. In surgery group, postoperative fever is defined as a fever > 38 °C on 2 consecutive postoperative days or above 39 °C on any 1 postoperative day. Postoperative pneumonia was defined as a newly developed infiltrates on the chest radiograph after surgery. In addition, postoperative complications requiring pharmacological treatment or surgical endoscopic radiologic interventions were described.

### Questionnaires

To collect the socio-demographic status including general health status, psychometric characteristics, and quality of life, we developed a structured self-administrated questionnaire. Concerning to the questionnaire, after acquisition written consent of this study, our survey was conducted at the waiting time for tests or to see their doctor. To prevent omission error, our researcher repeatedly asked responders to fill in missing questions as much as possible. The socio-demographic questionnaire consisted of 12 items; age (at enrollment and at treatment), sex, status of employment (full or part-time, unemployed, housewife, or student), living with partner (yes or no), religion (present or absent), education level (middle school graduate or below, high school graduate, or college graduate or above), income (<200 Korea Won (KRW), 200–400 KRW, > 400 KRW), current smoking, alcohol drinking, current Eastern Cooperative Oncology Group (ECOG) performance status, kinds and the number of co-morbidities, and family history of gastric cancer (yes or no).

To assess psychometric characteristics, we used the standard questionnaire forms of the Hospital Anxiety and Depression Scale (HADS), Distress Thermometer, Type D personality scale, Connor-Davidson resilience scale, Pittsburgh sleep quality index, and Cancer Worry Scale.

HADS is a frequently used tool to assess psychological distress, which consists of 7 items for the anxiety and 7 items for the depression subscales^26^. The score was interpreted as normal, 0–7 points; borderline, 8–10 points; and abnormal, 11–21 points^27^. Internal consistency measured by Cronbach’s alpha of the HADS Korean version was reported as 0.89 for anxiety and 0.86 for depression^28^.

The Distress Thermometer consists of single item asking subjects to rate their distress in the past week using a visual analog scale. Developed by the National Comprehensive Cancer Network, it is an effective screening tool for detecting distress among patients with various diseases, such as cancer. They suggests that a score of 4 or higher on the Distress Thermometer indicates a clinically significant distress level^29^.

We used the 14-item Type D personality scale to assess for distressed personality. It consists of 7 items on negative affectivity and 7 items on social inhibition. Patients were categorized as Type D personality using a standardized cutoff score of ≥10 on both the negative affectivity and social inhibition subscales^30^. Internal consistency, measured by Cronbach’s alpha, was 0.86 for negative affectivity and 0.90 for social inhibition^31^.

To measure resilience, we used the Korean version of the 25-item Connor-Davidson resilience scale, which has good reliability and validity. Each item was rated on a 5-point Linkert scale from 0 (not true at all) to 4 (true nearly all the time), with higher scores reflecting greater resilience. The reported Cronbach’s alpha values of the adapted Korean version was 0.93^32^.

To measure sleep quality, we used the Pittsburgh sleep quality index, which is a self-rated questionnaire assessing sleep quality and disturbances over a 1-month time interval^33^. Nineteen items compose 7 components: sleep quality, sleep latency, sleep duration, habitual sleep efficiency, sleep disturbances, use of sleeping medication, and daytime dysfunction^33^. The Cronbach’s α coefficient for internal consistency was 0.84, which shows high reliability^34^.

To measure worry about cancer recurrence, we used the validated Cancer Worry Scale questionnaire. We formally granted permission to use the Functional Assessment of Cancer Therapy – Gastric Cancer (FACT-Ga) by the copyright owner. It contains 13 items measuring 4 subjective aspects of worry: judged risk of cancer, worry about cancer recurrence, general worry about cancer over the previous months, and symptom level. A higher score indicates a greater concern about cancer recurrence. In our study, the Cronbach’s alpha value of the adapted Korean version was 0.90.

QoL was assessed using the Korean Version of FACT-Ga, version 4. We formally granted permission to use the FACT-Ga by the copyright owner. FACT-Ga has developed as a GC-specific instrument to evaluate the patients’ QoL. FACT-Ga consists of FACT-G (28 items) and a gastric cancer subscale (GCS, 19 items). FACT-G comprises four general subscales: physical well-being (PWB, 28 items), social well-being (SWB, 28 items), emotional well-being (EWB, 24 items), and functional well-being (FWB, 28 items). GCS consists of 19 items for assessment of patient-reported general symptoms (4 items; weakness, fatigue, loss of weight, and loss of appetite), gastrointestinal symptoms (8 items: trouble swallowing, reflux or heartburn, abdominal pain or discomfort in the stomach area, pain or discomfort while eating, fullness or heaviness in the stomach area, swelling or cramping in the stomach area, diarrhea, and gas or flatulence), and lifestyle changes related to gastrointestinal function (7 items; change of eating habits, enjoying meals with family or friends, eating favorite foods, avoidance of eating-out, interference with usual activities, worry about stomach problems, difficulty in planning for the future due to illness). The aggregate of PWB, SWB, EWB, FWB, and GCS scores is the FACT-Ga total. Therefore, the higher score of FACT-Ga could be reflecting higher QoL. The internal consistency of each subscale was tested by calculating the Cronbach’s alpha coefficients. In our study, all subscales demonstrated good internal consistency with α > 0.8 (0.897, 0.884, 0.887, 0.875, 0.894, and 0.854; Cronbach’s alpha coefficients of PWB, SWB, EWB, FWB, GCS, and FACT-Ga total, respectively).

### Sample size calculation

For recruitment of enough subjects to achieve sufficient statistical power, we calculated the sample size based on the previous study of the comparison of QoL between ESD and gastrectomy groups using G*Power Version 3.0.10 software program (Franz Fual, University of Kiel, Germany)^10^. With the total sample size of 434 subjects (217 subjects in each group), we would achieve the 80% power at the 0.05 significance level to detect a mean difference of 0.5 points in QoL between the ESD and gastrectomy groups, assuming that QoL would be higher in the patients with ESD group. Considering the dropout rate of 10%, a total of 482 subjects (241 subjects per each group) would be needed to detect significant outcomes.

### Statistical analyses

In the comparison of all variables between the ESD and surgery groups, continuous values were presented as means ± standard deviations, while categorical variables were shown as proportions. Statistical differences were determined using the *t*-test and chi-square test for continuous and categorical variables, respectively.

We performed an analysis of covariance (ANCOVA) to investigate whether there are significant differences in the QoL subscale scores between the two groups after adjusting for various variables and factors. ANCOVA Model 1 was adjusted by the age at enrollment and sex. ANCOVA Model 2 was adjusted by age at enrollment, sex, years since treatment, education level, alcohol drinking, current ECOG performance status, number of co-morbidities, Distress Thermometer, and doctors’ recurrence worry in Cancer Worry Scale. All factors used for adjustment in Model 2 met *p* value < 0.1 in the univariate analyses. Prior to modeling, we confirmed that the variance inflation factor values of these variables were less than 2. We reported the results as least square means with 95% confidence interval (CI) in ANCOVA Models 1 and 2. Additionally, we tested the elapsed time × group interaction to determine whether the QoL’s difference of GCS between two groups constantly continues over time.

Statistical analyses were performed using SPSS ver. 20.0 for Windows (SPSS Inc., Chicago, IL, USA), and *p* values < 0.05 were considered statistically significant.

## References

[1] Jung KW, Won YJ, Kong HJ, Lee ES (2018). Cancer Statistics in Korea: Incidence, Mortality, Survival, and Prevalence in 2015. Cancer Res Treat.

[2] Suh M (2017). Trends in Participation Rates for the National Cancer Screening Program in Korea, 2002–2012. Cancer Res Treat.

[3] Korean Gastric Cancer Association Nationwide Survey on Gastric Cancer in 2014. *J Gastric Cancer* **16**, 131–40 (2016). 10.5230/jgc.2016.16.3.131 PMC5065942 27752390

[4] Nunobe S, Hiki N (2017). Function-preserving surgery for gastric cancer: current status and future perspectives. Transl Gastroenterol Hepatol.

[5] Misawa K (2015). Long-term quality of life after laparoscopic distal gastrectomy for early gastric cancer: results of a prospective multi-institutional comparative trial. Gastric Cancer.

[6] Tanabe S (2017). Long-term outcomes of endoscopic submucosal dissection for early gastric cancer: a multicenter collaborative study. Gastric Cancer.

[7] Sumiyoshi T (2017). Short- and long-term outcomes of endoscopic submucosal dissection for early gastric cancer in elderly patients aged 75 years and older. Gastric Cancer.

[8] Fujishiro M (2017). Updated evidence on endoscopic resection of early gastric cancer from Japan. Gastric Cancer.

[9] Probst A (2017). Endoscopic submucosal dissection for early gastric cancer: are expanded resection criteria safe for Western patients?. Endoscopy.

[10] Choi JH (2015). Comparison of quality of life and worry of cancer recurrence between endoscopic and surgical treatment for early gastric cancer. Gastrointest Endosc.

[11] Kim YW (2008). Improved quality of life outcomes after laparoscopy-assisted distal gastrectomy for early gastric cancer: results of a prospective randomized clinical trial. Ann Surg.

[12] Zhang JK (2016). Type D Personality in Gastric Cancer Survivors: Association With Poor Quality of Life, Overall Survival, and Mental Health. J Pain Symptom Manage.

[13] Carless D, Douglas K, Fox K, McKenna J (2006). An alternative view of psychological well-being in cardiac rehabilitation: Considering temperament and character. Eur J Cardiovasc Nurs..

[14] Mols F, Thong MSY, de Poll-Franse LVV, Roukema JA, Denollet J (2012). Type D (distressed) personality is associated with poor quality of life and mental health among 3080 cancer survivors. J Affect Disord.

[15] Yoon HS (2015). Short Sleep Duration and Its Correlates among Cancer Survivors in Korea: the Korea National Health and Nutrition Examination Surveys. Asian Pac J Cancer Prev.

[16] Harms CA (2019). Quality of life and psychological distress in cancer survivors: The role of psycho-social resources for resilience. Psychooncology.

[17] Skarstein J, Aass N, Fossa SD, Skovlund E, Dahl AA (2000). Anxiety and depression in cancer patients: relation between the Hospital Anxiety and Depression Scale and the European Organization for Research and Treatment of Cancer Core Quality of Life Questionnaire. J Psychosom Res.

[18] Aminisani N, Nikbakht H, Asghari Jafarabadi M, Shamshirgaran SM (2017). Depression, anxiety, and health related quality of life among colorectal cancer survivors. J Gastrointest Oncol..

[19] Han KH (2013). Factors associated with depression in disease-free stomach cancer survivors. J Pain Symptom Manage..

[20] Kim YI (2018). Serial intermediate-term quality of life comparison after endoscopic submucosal dissection versus surgery in early gastric cancer patients. Surg Endosc..

[21] Yu W, Park KB, Chung HY, Kwon OK, Lee SS (2016). Chronological Changes of Quality of Life in Long-Term Survivors after Gastrectomy for Gastric Cancer. Cancer Res Treat.

[22] Avery K (2010). Health-related quality of life and survival in the 2 years after surgery for gastric cancer. Eur J Surg Oncol.

[23] Karanicolas PJ (2013). Quality of life after gastrectomy for adenocarcinoma: a prospective cohort study. Ann Surg.

[24] Kim SG (2017). Quality of Life after Endoscopic Submucosal Dissection for Early Gastric Cancer: A Prospective Multicenter Cohort Study. Gut Liver.

[25] Paner GP (2018). Updates in the Eighth Edition of the Tumor-Node-Metastasis Staging Classification for Urologic Cancers. Eur Urol..

[26] Snaith RP (2003). The Hospital Anxiety And Depression Scale. Health Qual Life Outcomes.

[27] Zigmond AS, Snaith RP (1983). The hospital anxeity and depression scale. Acta psychiatrica Scandinavica.

[28] Oh SM, Min KJ, Park DB (1999). A study on the standardization of the hospital anxiety and depression scale for Koreans-A comparison of normal,depressed and anxious group. J Korean Neuropsychiatr Assoc..

[29] National Comprehensive Cancer Network (2010) Clinical practice guidelines in oncology-v.1.2010. Distress management: version1.

[30] Denollet J (2005). DS14: standard assessment of negative affectivity, social inhibition, and Type D personality. Psychosom Med.

[31] Lim HE (2011). Assessment of the type D personality construct in the Korean population: a validation study of the Korean DS14. J Korean Med Sci.

[32] Jung YE (2012). The Korean version of the Connor-Davidson Resilience Scale: an extended validation. Stress Health..

[33] Buysse DJ, Reynolds CF, Monk TH, Berman SR, Kupfer DJ (1989). The Pittsburgh Sleep Quality Index: a new instrument for psychiatric practice and research. Psychiatry Res..

[34] Sohn SI, Kim DH, Lee MY, Cho YW (2012). The reliability and validity of the Korean version of the Pittsburgh Sleep Quality Index. Sleep Breath..

